# Trophic transfer and egestion dynamics of microplastics in the *Brachionus-Asplanchna* rotifer system

**DOI:** 10.1007/s10661-026-15485-w

**Published:** 2026-05-22

**Authors:** Jorge Jiménez-Contreras, Alejandro Salvador-Martínez, Marco Antonio Jiménez-Santos, Michael Anai Figueroa-Sánchez, Alfonso Lugo-Vázquez, Mario Alfredo Fernández-Araiza

**Affiliations:** 1 Laboratorio de Producción Acuícola, FES Iztacala, Universidad Nacional Autónoma de MéxicoCol. Los Reyes IztacalaEstado de México, Av. De los Barrios 1, 54090 Tlalnepantla, CP Mexico; 2 Limnoterrestrial Ecology Lab, Institute of Soil Biology BC CAS, České Budějovice, Czech Republic; 3 Laboratory of Non-Mendelian Evolution, Institute of Animal Physiology and Genetics CAS, Liběchov, Czech Republic; 4 Department of Ecosystem Biology, Faculty of Science, University of South Bohemia, Branišovská 1760. 37005, České Budějovice, Czech Republic; 5 Grupo de Investigación en Limnología Tropical, UIICSE, FES Iztacala, Universidad Nacional Autónoma de México Col. Los Reyes IztacalaEstado de México, Av. De los Barrios 1, 54090 Tlalnepantla, CP Mexico

**Keywords:** Bioaccumulation, Biomagnification, Anthropogenic Stressors, Emerging Contaminants, Rotifers, Experimental Ecotoxicology

## Abstract

Microplastics have become a pervasive contaminant in freshwater ecosystems worldwide, raising concerns about their impact on aquatic life, especially zooplankton, which play a pivotal role in freshwater food webs. Despite their ecological importance, knowledge on how microplastics move through predator–prey interactions in microscopic communities remains limited. We investigated this question using a classic freshwater system: the filter-feeding rotifer *Brachionus caudatus* and its predator, *Asplanchna brightwellii*. Specifically, we asked: How efficiently do these organisms ingest and eliminate microplastics, and to what extent are the particles transferred across trophic levels? We exposed *B. caudatus* to three concentrations of 1 µm fluorescent polystyrene microspheres (0.1, 0.5, and 1.0 µg ml^−1^) for 24 h, then fed this contaminated prey to *A. brightwellii* predators. Using fluorescence microscopy, we tracked the fate of the microplastics over 48 h in both species. Both rotifers readily consumed the microspheres, and they also eliminated them efficiently. Fluorescence intensity declined rapidly in both prey and predator, with minimal retention after 24–48 h. While predators initially accumulated more than twice the fluorescence of their prey (confirming trophic transfer), this accumulation was short-lived. The rapid clearance suggests that in this simple food chain, 1 µm polystyrene microspheres do not biomagnify. Our findings reveal that these microplastics behave more like transient contaminants than persistent pollutants in rotifer communities, offering new insights into how microplastics flow through freshwater food webs at the microscopic scale.

**Supplementary Information:**

The online version contains supplementary material available at 10.1007/s10661-026-15485-w.

## Introduction

The term "emerging pollutants" refers to chemical compounds commonly found in water at trace concentrations (ranging from ng L^−1^ to µg L^−1^) that are not easily detectable in routine monitoring (Norman -Network, 2025; Geissen et al., 2015). These contaminants can cause alterations in water quality, the physiology of organisms, and their homeostatic processes (Annamalai & Namasivayam, 2015). Emerging contaminants include drugs, heavy metals, and pesticides, as well as hormones, industrial additives, microbeads, and microplastics (Coronado-Apodaca et al., 2023; Geissen et al., 2015; Rodriguez-Narvaez et al., 2017). Among these, microplastics (MPs) have gained prominence because of their extreme persistence, global dispersal, and capacity to act as vectors for other contaminants (Bexeitova et al., 2024; Nava et al., 2023).

Microplastics (< 5 mm), have become a ubiquitous contaminants throughout the biosphere (Wagner & Lambert, 2018; Wagner et al., 2014); their low density, high surface area, and resistance to degradation facilitate atmospheric and fluvial transport into diverse environments, including epicontinental water bodies (Dris et al., 2016; Rochman, 2018). In such systems, concentrations have been reported from 1.2 × 10^–3^ to 542,000 items m^3^ (Baldwin et al., 2016; Lu et al., 2021; Yardy et al., 2022). Most particles are chemically identified as polyethylene (PE) in high frequency, polystyrene (PS), and polypropylene (PP) (De Sá et al., 2018; Koelmans et al., 2019; Parker et al., 2021; Zhao et al., 2022). Their widespread distribution and high concentration in water bodies make them potentially toxic to freshwater biota, especially zooplankton, which, due to their key position in the food web and their multiple feeding strategies, are vulnerable (Scherer et al., 2018).

Microplastic ingestion by zooplankton has been documented in situ, with reported values ranging from 0.002 to 0.2 items ind^−1^ in rotifers, cladocerans, and copepods (Jiménez-Contreras et al., 2024; Sánchez-Campos et al., 2024). However, most studies on microplastic consumption in zooplankton have been conducted in laboratory assays (Phuong et al., 2016). These studies have involved model species such as cladocerans of the genus *Daphnia* (Yin et al., 2023), copepods (DeMott, 1986), and mainly in rotifers of the genus *Brachionus* (Jeong et al., 2016; Xue et al., 2021). Despite this progress, trophic transfer of MPs across zooplanktonic predator–prey links is still scarce. For example, Galir et al. (2025) analyzed the effect of microplastics on the grazing and mortality of freshwater zooplankton, finding reduced grazing activity and increased mortality when zooplankton were exposed to smaller microplastics (1–5 µm). On the other hand, mesocosm experiments have demonstrated the movement of MPs from primary consumers to higher trophic levels such as predators (Yıldız et al., 2022).

Primary-consumer rotifers such as *Brachionus* often co-occur with predatory *Asplanchna* spp. (Wallace et al., 2006). In Mexico, 22 species of the genus *Brachionus*, and eight species of the Asplanchnid predators have been recorded (Sarma et al., 2021). The diet of species of *Asplanchna* includes protozoans, other rotifers, and small cladocerans, particularly in field observations, where they consume prey of the Brachionidae family can even be depleted in a short time (Chang et al., 2010; Nandini et al., 2003; Sarma & Nandini, 2007; Sarma et al., 2007). Because of this, such predator–prey interactions in rotifers, as in other freshwater communities, are of great relevance due to their strong influence on community structure given their wide distribution and high abundances (Dumont et al., 2012). The *Asplanchna-Brachionus* system has been successfully used in laboratory tests to analyze the effect of various toxins such as heavy metals (Gama-Flores et al., 2007), pesticides (Gómez et al., 1997), and, more recently, microplastics (Hernández-Lucero et al., 2024). Using this model, it is possible to examine whether MPs ingested by prey are subsequently transferred to predators and assess potential bioaccumulation or biomagnification within short freshwater food chains.

The present study therefore investigates (i) the ingestion and egestion dynamics of 1 µm fluorescent PS spheres in the filter-feeder *B. caudatus* and the predator *A. brightwellii*, and (ii) the efficiency of trophic MP transfer between these rotifer species under controlled laboratory exposure scenarios. Our findings provide novel insight into MP flux through rotifer-based trophic links, a key but understudied pathway in freshwater ecosystems, and contribute data for future risk assessments and ecological models.

## Materials and methods

### Rotifer culture

The rotifers *Asplanchna brightwellii* and *Brachionus caudatus* were isolated from a zooplankton sample in the littoral zone of Lake Xochimilco, Mexico (19.25310° N, 99.09283° W) and maintained under laboratory conditions for 6 months prior to experimentation. Cultures were maintained in 1 L glass beakers filled with synthetic, moderately hard EPA (1.92 g NaHCO_3_, 1.2 g CaSO_4_, 1.2 g MgSO_4_, and 0.08 g KCl in 20 L of deionized water) (US-EPA, 2002). The filter-feeding rotifer *B. caudatus* was fed with green algae *Tetradesmus obliquus* (1.0 × 10^6^ cells ml^−1^), whereas the predator *A. brightwellii* received a mixed diet of brachionids. Both cultures were maintained at 25 ± 1 °C under a 16:8 h light:dark photoperiod (Pérez-Legaspi & Rico-Martínez, 1998). *T. obliquus* was grown in Bold's basal medium (Borowitzka & Borowitzka, 1988), harvested in logarithmic growth phase, centrifuged at 3000 rpm for five minutes, resuspended in distilled water, and cell concentration was determined with a hemocytometer prior to use.

### Test particles

Throughout the experiments, 1 µm fluorescent polystyrene spheres (Thermo Fisher Scientific; excitation 430 nm, emission at 465 nm, density of 1.05 g cm^−3^, and a stock concentration 1 × 10^10^ items ml^−1^) were used as MPs to quantify ingestion and egestion. This particle type was selected because its size and buoyancy favor persistence in the water column and may increase the probability of biological translocation (Liang et al., 2021; Yardy et al., 2022; Zhao et al., 2022). The concentrations used in this study were selected to establish controlled laboratory exposure conditions for evaluating short-term ingestion and egestion dynamics. Before each use, all MP suspensions were vortexed to prevent aggregation. No chemical dispersants were added to the suspensions to avoid introducing confounding toxicological variables.

### Ingestion and egestion of MPs

The microplastic ingestion and egestion experiments were conducted in two phases: the first with the prey (*B. caudatus*), the results of which were used to determine the optimal exposure conditions for the second phase with the predator (*A. brightwellii*).

### Phase I – prey exposure

The rotifers *B. caudatus* were exposed to three independent MP concentrations (0.1, 0.5, and 1.0 µg ml^−1^). The MP solutions were prepared from the stock by dilution in EPA medium followed by vortexing. Rotifers (10 ind. ml^−1^) were exposed to MPs for 24 h at 25 °C in darkness with *T. obliquus* as food (0.5 × 10^6^ cells ml^−1^). This exposure was conducted in darkness (Jeong et al., 2016, 2018) primarily to prevent the photodegradation and fluorescence loss of the microparticles. Furthermore, any potential physiological effects induced by darkness on the prey are negligible within this short experimental time frame. After exposure, rotifers were filtered through a 50 µm mesh and washed using EPA medium to remove any remaining free particles. To track egestion, fifty individuals were transferred to 10 ml EPA medium (with food) for each ingestion time (post-exposure interval 0, 2, 4, 24, and 48 h). At each interval, rotifers were rinsed, fixed with 4% formaldehyde in 1.5 ml Eppendorf tubes, and stored at 4 °C in the dark until observation. Control groups of 50 individuals were fed only with *T. obliquus* for 24 h and subsequently fixed and placed in Eppendorf tubes. Ten individuals (n = 10 replicates) per replicate were randomly selected for microscopy and image recording. Fluorescence intensity was quantified in 10 ind. for each MP concentration and post-exposure interval resulting in a total of 180 rotifers included in the analysis. Fluorescence photographs were obtained using a Zeiss Scope A.1 microscope under the same UV light power level. Image parameters were subsequently homogenized, and fluorescence was quantified with ImageJ 1.53 K following the standard protocols and user manual for fluorescence microscopy image analysis (Bankhead, 2014; Manabe et al., 2011; Schneider et al., 2012).

### Phase II – predator exposure

To analyze MP ingestion in the predatory rotifer *A. brightwellii*, the lowest concentration (0.1 µg ml^−1^) to which prey was exposed was selected. At this concentration, individuals exhibited detectable fluorescence and no apparent effect on their mobility, allowing the predator to detect and ingest them normally (lowest effective concentration). To evaluate trophic transfer, a total of 1,200 *B. caudatus* individuals were first exposed to 0.1 µg ml⁻^1^ MPs under the conditions described above. Subsequently, adult *A. brightwellii* predators (previously subjected to a 4-h starvation period) were fed with these treated prey for 2 h at a density of 5 ind. ml⁻^1^, which is optimal to facilitate predator–prey encounters (Sarma et al., 2007). Following this 2 h feeding window, ten asplanchnids per time point (n = 10 replicates) were individually transferred to a clean medium (without microplastics) containing a lower density of uncontaminated prey (1 ind. ml⁻^1^) to keep them active while evaluating egestion. This predator–prey interaction phase was also conducted in darkness to preserve particle fluorescence. A brief 2 h feeding window at an optimal prey density was established, as short-term ingestion in these non-visual predators is unaffected by light absence (Sawada & Enesco, 1984). Asplachnid rotifers were collected at 0, 4, 8, 24, and 48 h, fixed in 4% formaldehyde, examined, and photographed under a fluorescence microscope. The fluorescence intensity was analyzed using ImageJ software (Manabe et al., 2011; Schneider et al., 2012).

## Statistical analysis

To assess the effects of exposure time and microplastic concentration on fluorescence intensity in Phase I of *B. caudatus*, we employed a generalized linear model (GLM) with a Gamma error distribution and a log link function. This approach was selected for its suitability in modelling continuous, positive, and right-skewed data (McCullagh & Nelder, 1989). The model included time, concentration, and their interaction as fixed factors:

$$log(\mu ij)=\beta 0+\alpha i+\gamma j+(\alpha \gamma )ij$$

where μ_ij_ is the expected fluorescence, α_i_ and γ_j_ represent the main effects of time and concentration, and (αγ)_ij_ represents their interaction term.

Model adequacy was verified through diagnostic plots of deviance residuals versus fitted values, which showed no systematic patterns or heteroscedasticity (Supplementary Information, Fig. S1). The Gamma distribution and log link function were chosen based on the empirical relationship between mean and variance, which increased proportionally with fluorescence intensity. The significance of main effects and their interaction was evaluated using likelihood ratio tests (Type III analysis of deviance) by comparing nested models. Pairwise post hoc comparisons were subsequently performed using Tukey's honestly significant difference (HSD) tests within each concentration level to identify significant differences among time points. The statistical analyses were performed in R (R Core Team, 2024); the script is available in the Supplementary Information.

To complement this parametric approach, we also performed a non-parametric Kruskal–Wallis test followed by Dunn's multiple comparison procedure, a classical method commonly used in ecotoxicological studies. Although both methods yielded consistent biological interpretations, the GLM provided a more robust and informative framework by explicitly modelling the interaction between time and concentration while accounting for variance heterogeneity. Results from the Kruskal–Wallis analysis are presented in the Supplementary Information (Fig. S2).

For Phase II (predator exposure), differences in the relative fluorescence of *A. brightwellii* across the egestion time points were analyzed using a non-parametric Kruskal–Wallis test followed by Dunn's multiple comparison procedure to identify significant differences between specific observation hours. This specific analysis was performed using SigmaPlot version 11.0.

## Results and discussion

**Fig. 1:**
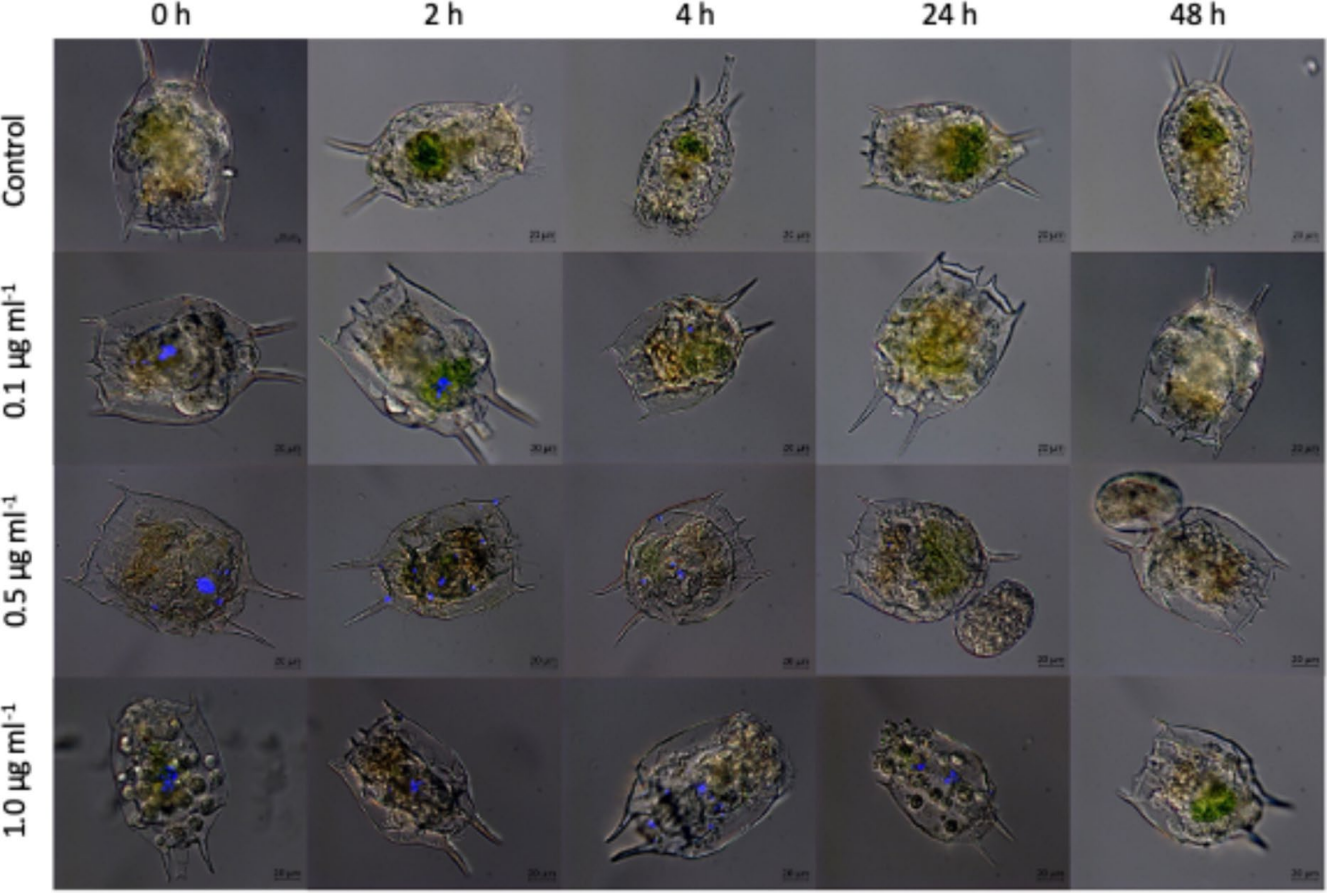
Merged micrographs (Bright light-Fluorescence) of 24 h consumption and ingestion of three concentrations (0.1, 0.5, and 1.0 µg ml^−1^) of fluorescently labeled MPs in *B. caudatus* over a 48 h period

**Fig. 2:**
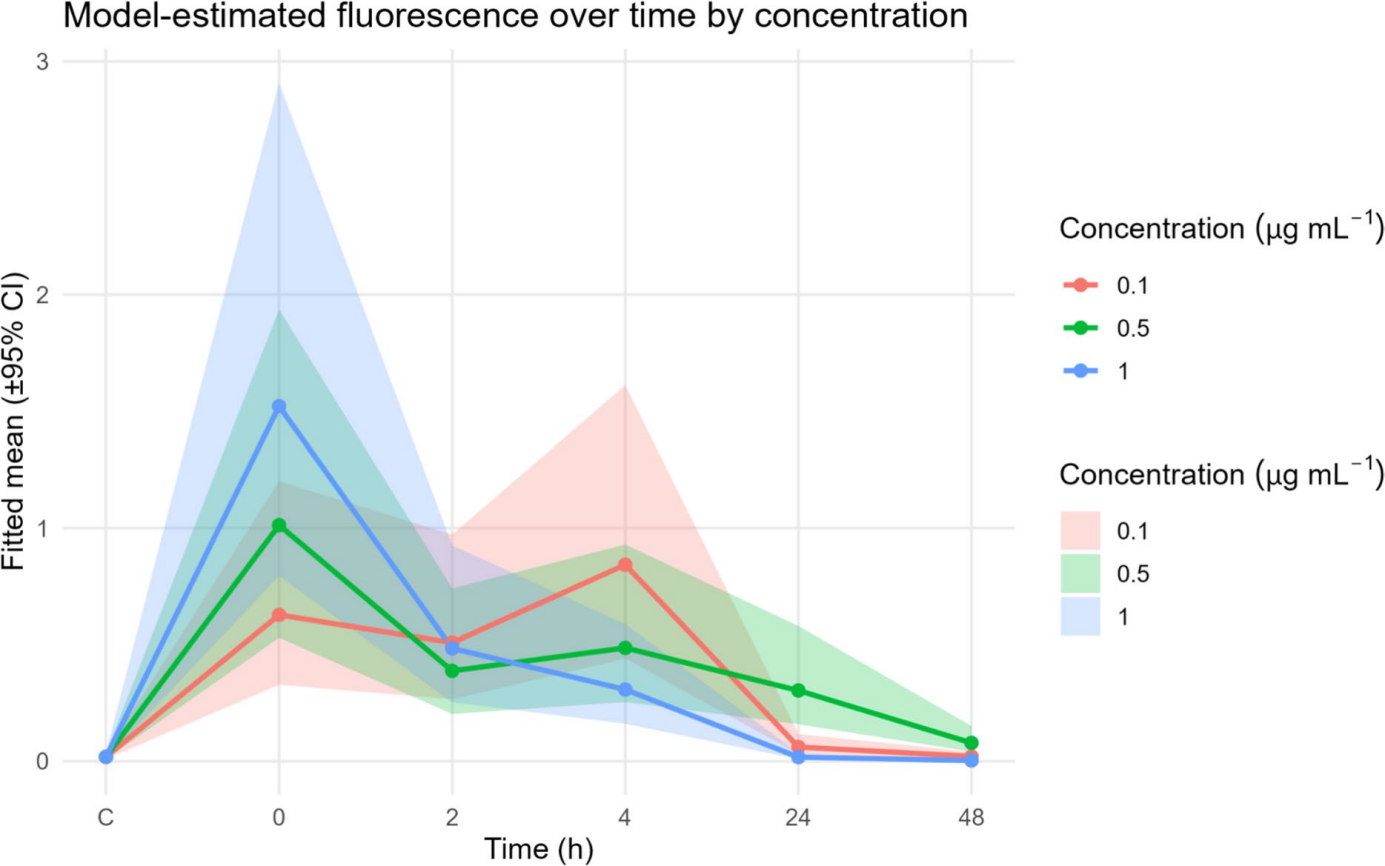
Model-estimated fluorescence over time by concentration. Temporal patterns of fluorescence at varying microplastic concentrations. Mean fluorescence predictions (with 95% confidence intervals) from a Gamma generalized linear model examining the combined effects of egestion time and microplastic concentration. The shaded areas show 95% confidence intervals around model estimates. C = denotes the control treatment

The Exposure of *Brachionus caudatus* to 1 µm fluorescent polystyrene microspheres (microplastics MP) revealed clear time and concentration-dependent ingestion patterns (Fig. 1, Fig. 2). Fluorescence was detected in all treatments, indicating active ingestion of MP particles by rotifers. Importantly, no mortality was recorded in either species across any of the treatments during the exposure and depuration periods. Although suspensions were initially homogenized by vortexing, the absence of chemical dispersants implies that some spatial heterogeneity or minor settling might naturally occur over the 24 h period. However, visual observations during the exposure did not indicate severe macroscopic aggregation, adhesion to the vessel walls, or massive trapping at the water surface. Furthermore, because the exposure was conducted in synthetic EPA medium rather than natural freshwater, rapid biogenic aggregation mediated by natural bacteria and exopolymers (Drago et al., 2020) was minimized. Combined with the active swimming behavior of *B. caudatus*, this ensured a continuous exposure scenario. At the beginning of the observation period post-exposure (0 h), fluorescence intensity was highest, and progressively decreased over time, reaching minimal levels after 48 h. The Gamma GLM revealed significant effects of exposure time and MP concentration, as well as their interaction (both time and interaction between time and concentration: p < 0.001, concentration p < 0.01; Table 1). The highest consumption of microplastics in terms of fluorescence was observed at 1.0 µg ml^−1^, while the lowest concentration was 0.1 µg ml^−1^, both at time zero. This pattern indicates rapid ingestion followed by efficient egestion, and minimal retention of MP in the digestive tract, consistent with previous studies on short-term microplastic turnover in zooplankton (Fueser et al., 2020; Jeong et al., 2016).

**Table 1:** Main results of the Gamma generalized linear model (GLM) evaluating the effects of exposure time and microplastic concentration on fluorescence intensity

	Chi^2^	gl	p-value
Interaction: Time × Concentration	67.133	10	< 0.001
Time	307.383	5	< 0.001
Concentration	18.549	2	< 0.01

Rotifers of the genus *Brachionus* are considered generalist suspension feeders (Gilbert & Bogdan, 2019), meaning they are capable of consuming algal cells of various sizes (preferably 1 and 10 µm), limited by the size of their oral structure (Gilbert & Bogdan, 2019; Monakov, 2003; Rothhaupt, 1990). This likely explains why the microparticles were taken up across all tested concentrations in this study. Furthermore, a study by DeMott (1986) that tested zooplankton for taste response in food selection showed that certain brachionids (*B. calyciflorus*) feed non-selectively, ingesting both "flavoured" polystyrene particles and polystyrene alone equally.

Our findings align with broader ecotoxicological research; the ingestion of microplastics by freshwater zooplankton has been documented through laboratory tests, verifying that two of the main groups studied, rotifers and cladocerans, can ingest particles from 0.05 to 30 µm (Jeong et al., 2016; Reyes-Santillán et al., 2024) and between 0.05 and 100 µm (Frankel et al., 2020; Rehse et al., 2016), respectively. In recent studies, Galir et al. (2025) analyzed the ingestion of three categories of microplastics (1–5 μm, 27–32 μm, and 45–53 μm) by freshwater zooplankton (rotifers, cladocerans, and copepods), observing a higher intake of smaller microplastics (1–5 μm) by all three taxonomic groups. Likewise, a direct relationship has been observed between microplastic consumption by rotifers and particle concentrations in the environment, which has also been linked to adverse effects on their demographic variables (Nugnes et al., 2022). In our short-term trophic transfer experiments (Phase II), we strategically utilized the lowest exposure concentration (0.1 µg ml⁻^1^) to ensure that the prey maintained natural swimming behaviors, as confirmed by direct observation during the assays. While evaluating the specific physiological effects of higher microplastic concentrations falls outside the scope of our study, we expect that higher levels would significantly alter the predator–prey dynamics. It is well recognized that elevated microplastic concentrations exert negative effects on rotifers; for instance, Sun et al. (2019) demonstrated that small-sized microplastics significantly decrease rotifer survival and reproduction. Therefore, maintaining a low exposure concentration was crucial in our design to observe trophic transfer without artificially modifying the prey's behavior and increasing their vulnerability to the predator.

Despite the observed and documented capacity for ingestion across rotifer species, the permanence of MPs in the digestive system remains a transient process. Previous studies indicate that microplastics have relatively short retention times (< 48 h) within the digestive tract of rotifers (Jeong et al., 2018; Snell & Hicks, 2011; Xue et al., 2021). Our results indicate relative fluorescence variations between 0.62 ± 0.19 at 0 h and 0.02 at 48 h in the 0.1 µg ml^−1^ treatment, between 1.01 ± 0.5 at 0 h and 0.07 at 48 h in the 0.5 µg ml^−1^ treatment, between 1.5 ± 0.28 at 0 h and 0.37 at 48 h in the 1.0 µg ml^−1^ treatment and the control group presented average fluorescence values below 0.01 ± 0.002.

In this study, across all three MP concentrations, significant differences in fluorescence were found (p < 0.001, Supplementary Information Table S1) between the controls and the first three times (0, 2, and 4 h); however, from 24 h onwards, no significant differences were found compared to the control. These results indicate that 24 h was the limit for the near-complete excretion of ingested MPs in our treatments. This rapid depuration is consistent with findings in other Brachionids, such as *B. koreanus* (Jeong et al., 2016) *B. plicatilis* (Lian et al., 2025), and *B. calyciflorus* (Xue et al., 2021), where MPs are excreted within 24 to 48 h, without apparent accumulation in other organs. However, Jeong et al. (2018) studied the accumulation of polystyrene microbeads (0.05, 0.5, and 6.0 µm) and their potential relationship with the increase in toxicity of Persistent Organic Pollutants (POPs), documented an inverse relationship between microbead size and persistence percentages at 24 h. They found that the smallest particles (0.05 µm) had the highest accumulation percentages in rotifers and could also be observed in areas outside the rotifer digestive tract, suggesting these microbeads may be membrane permeable. In our species, *B. caudatus*, the absence of visible particle aggregation or retention structures suggests that the smooth surface and small size of the microspheres (1 µm) reduce adhesion to gut epithelia. This mechanical property likely contributes to the transient retention of MPs and explains the observed fluorescence decline. It is important to note, however, that our experimental design evaluated microplastic ingestion and elimination in the continuous presence of organic food (*T. obliquus*), aiming to simulate ecologically relevant feeding conditions. This constant influx of organic matter may mechanically facilitate the rapid flushing of transient microparticles from the gut. Comparable findings in crustaceans (Zhang et al., 2023) and nematodes (Fueser et al., 2020) demonstrate that smaller, spherical MPs particles are excreted more efficiently than irregular or fibrous ones.

Although direct baseline measurements of the transit time for natural food were not recorded in our control groups, a limitation that future experiments should address, published gut-passage rates provide a vital baseline. It has been documented that the gut passage time (GPT) for food particles varies between 15 and 30 min in *Brachionus* species (Lindemann & Kleinow, 2000; Starkweather & Gilbert, 1977), while complete gut filling occurs within 12 h (Walford & Lam, 1987; Watanabe, 1993). For predators like *A. brightwellii*, baseline feeding studies indicate that several hours of continuous exposure are required to fully evaluate prey ingestion and gut transit (Sawada & Enesco, 1984). Regarding microplastics, quantitative studies estimate that a *Brachionus* rotifer can consume up to 59 beads min^−1^ (1.6 µm MPs) and excrete 95% of them within 120 min (Baer et al., 2008). Considering that the digestive tract of a brachionid has a volume between 60,000 and 120,000 µm^3^ (Kleinow et al., 1991) and our 1 µm microspheres have an approximate volume of 0.524 µm^3^, rotifers could theoretically hold over 200,000 spheres when full. Excreting such a massive number of particles logically requires a longer timeframe, which explains the 24 to 48 h needed for complete egestion in our study. Prolonged residence times of particles within the gut can negatively affect feeding rates and assimilation efficiency (Salt, 1987).

Furthermore, the concentration of available organic food plays a critical role in modulating this microplastic transit and depuration. As noted by Drago et al. (2020), microplastic consumption in natural environments occurs in the presence of food, and adding microalgae to exposure treatments does not necessarily reduce the microplastic intake rate. In our exposure phase, *B. caudatus* was maintained with an algal concentration of 0.5 × 10⁶ cells ml^−1^ of *T. obliquus*, representing a saturating density that promotes continuous filtration and maximum intrinsic growth (Reyes-Santillán et al., 2024). Under these abundant food conditions, the constant ingestion of algal cells into the gut exerts a mechanical push on the vacuolar contents, accelerating the GPT for organic food—which can be as short as 20 min under high food availability—and enhancing the rapid egestion of microplastics via superfluous feeding (Haney et al., 1986; Hansen et al., 1997; Starkweather, 1980). Conversely, lower algal densities (e.g., < 1 × 10^5^ cells ml⁻^1^), common in fluctuating natural environments, induce an energy-limited state where rotifers maximize their clearance rate. This not only increases the probability of non-selective microplastic capture but also prolongs the GPT (e.g., 30–45 min) to maximize nutrient absorption, elevating the risk of mechanical irritation or chemical leaching (Drago & Weithoff, 2021; Xue et al., 2021). Therefore, the rapid clearance observed in our study is likely driven by the optimal food supply.

Beyond physiological capacity and transit times, these quantitative consumption and rapid depuration rates have significant implications for the ecological partitioning of microplastics. To fully understand what fraction of ingested particles ascend the food web versus what is removed from the water column, quantitative partitioning studies evaluating particle ingestion under varying environmental conditions are essential (Drago & Weithoff, 2021; Drago et al., 2020). Because rotifers egest particles rapidly and in large quantities, a substantial portion of these microplastics is likely packaged into fecal pellets or biogenic aggregates. As highlighted by related studies, such biological packaging alters particle behavior and accelerates their sedimentation rates, promoting benthic deposition rather than indefinite suspension. Consequently, while rotifers act as temporary pelagic carriers that facilitate trophic transfer to higher consumers (e.g., *A. brightwellii*), their high egestion rates also act as a biological pump, effectively removing a fraction of microplastics from the water column.

**Fig. 3:**
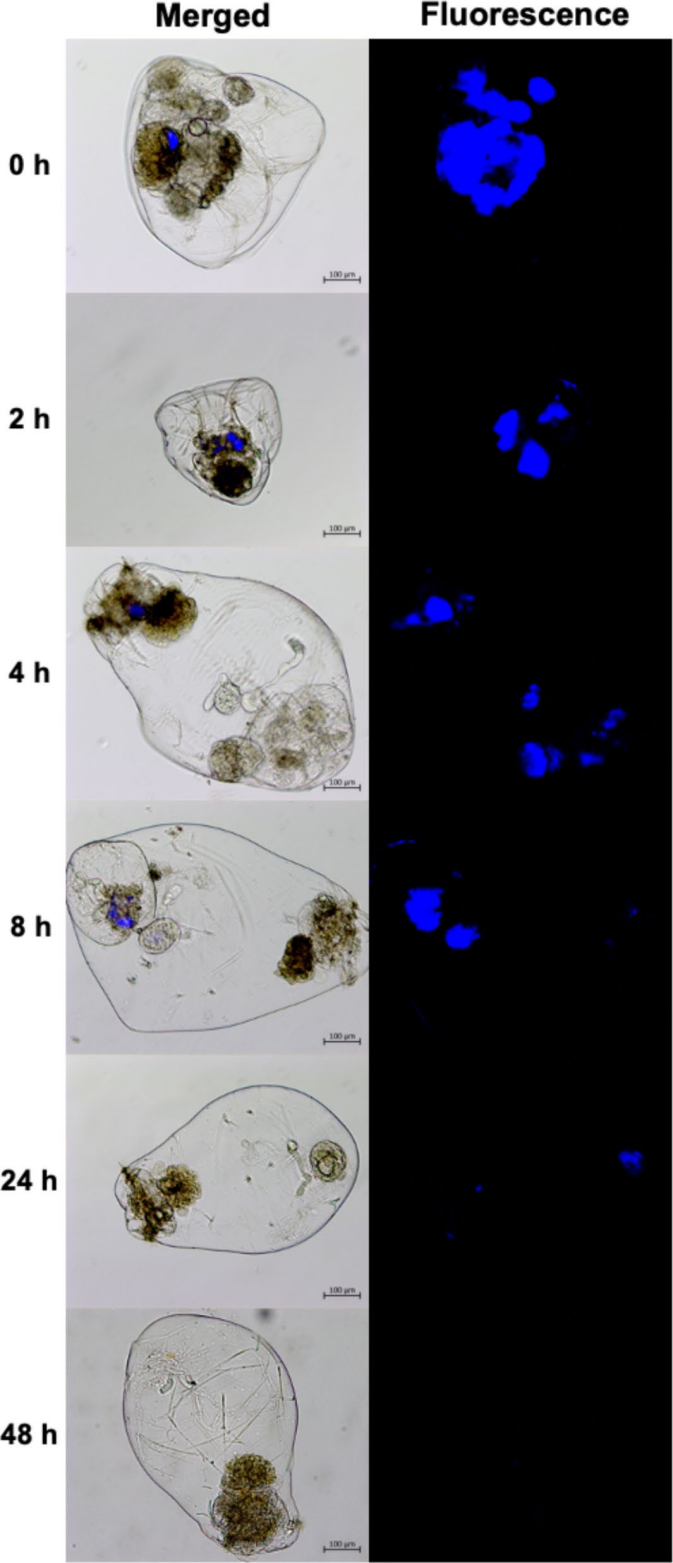
Micrographs of ingestion of fluorescently labeled MPs in *A. brightwellii*, ingested via prey (*B. caudatus*) exposed to MPs

**Fig. 4:**
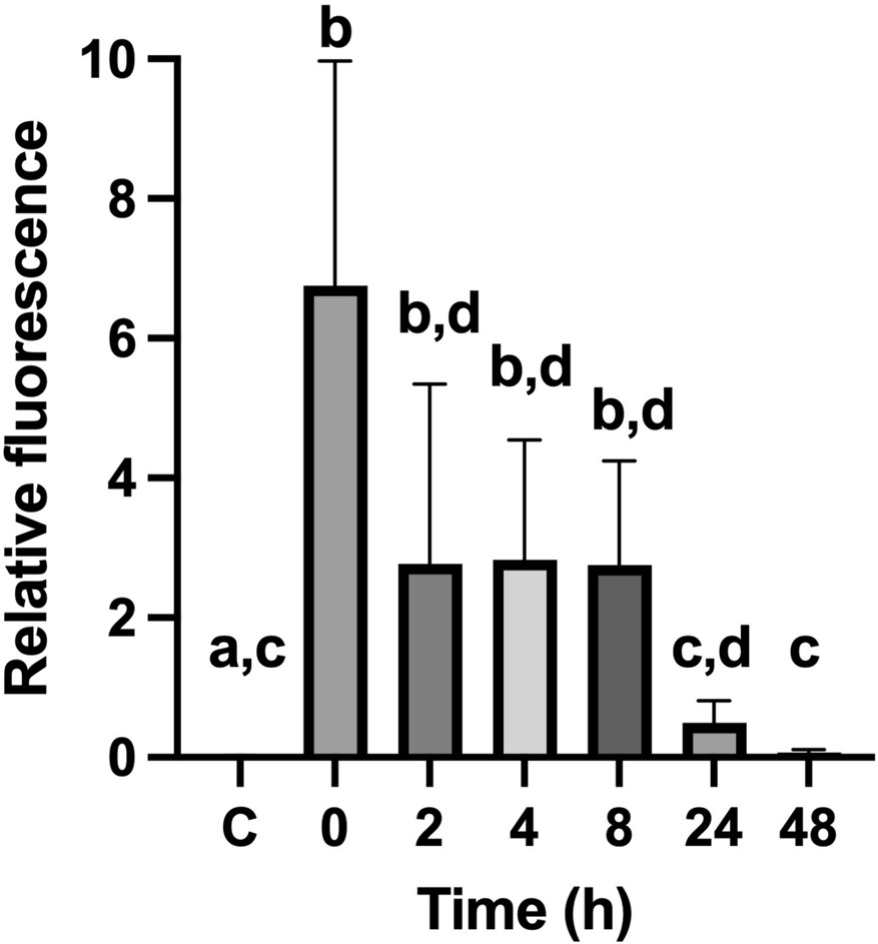
Changes in relative fluorescence during the total depuration time (48 h) of the predator *A. brightwellii* fed with prey exposed to 1 µm MPs at a concentration of 0.1 µg ml^−1^. C = Control group, prey without MPs

In ecotoxicology, single-species tests are frequently used, but their results could be limited for interpretation at higher levels of biological organization (Forbes, 1993; Rand & Petrocelli, 1985). Therefore, for the evaluation of ecological risks posed by contaminants, it is necessary to evaluate ecological interactions, such as competition or predation (Schmitt-Jansen et al., 2008). In the case of MPs, the need to include more than one species in ecotoxicological tests has been highlighted (De Sá et al., 2018) due to their potential biomagnification (the sequential increase in contaminant concentration across trophic levels) (Bhatt & Chauhan, 2023). In this sense, our results indicate that in the case of the rotifer predator *Asplanchna brightwellii*, greater relative fluorescence was observed at 0 h and a gradual decrease over time (Fig. 3). Relative fluorescence values ranged from 6.75 ± 1.01 at 0 h to 0.069 ± 0.01 at 48 h (Fig. 4). Significant differences (ANOVA, p < 0.05) were observed between the control and the 0, 2, 4, and 8 h treatments; however, no significant differences were observed with respect to the control after 24 h. Our data showed a more than twofold increase in fluorescence between the primary consumer *B. caudatus* and the predator *A. brightwellii*, confirming the transfer of MPs between these two trophic levels. While this higher internal particles load during feeding could be described as a form of transient or gastrointestinal biomagnification (sensu Setälä et al., 2014), *A. brightwellii* eliminated most of the ingested particles within two days.

Therefore, to determine the systemic biomagnification of a contaminant, its bioaccumulation (the uptake and net retention of a substance within an organism’s tissues from all environmental sources) at a given trophic level must first be verified (Edo et al., 2024; Streit, 1992). To date, the bioaccumulation of MPs in freshwater organisms has not been definitively proven (Bhatt & Chauhan, 2023); however, it can be inferred that MPs tend to bioaccumulate depending on their character and particle size (McIlwraith et al., 2021; Setälä et al., 2014). Our results show a decrease in fluorescence to practically zero, indicating the efficient elimination of MPs in both prey and predators within 48 h. This suggests that bioaccumulation of polystyrene microspheres did not occur in these rotifers at these tested concentrations and particle sizes. According to some authors (Galir et al., 2025; Jeong et al., 2018), the highest retention, translocation, and bioaccumulation of MPs in rotifers occurs with particles smaller than 5 µm, however, in our study using 1 µm particles, they were almost entirely excreted within 48 h. This behavior can be explained by the filtering physiology of rotifers and the hydrophobic, non-bioadhesive properties of PS microspheres. In contrast, irregular or fibrous particles often exhibit longer residence times and can mechanically interfere with digestion (Zhang et al., 2023). Hence, particle morphology and surface characteristics are critical determinants of ingestion-egestion kinetics. In works carried out in marine systems (Gao et al., 2024), the biomagnification of MPs has been documented using stable isotopes as markers in fish. However, in epicontinental systems with invertebrates, data are still scarce. For example, in microcosms, the transfer of microplastics to higher trophic levels has been verified in meiofauna (Fueser et al., 2020), and Yıldız et al. (2022) using microcosms confirmed the transfer of MPs to higher trophic levels using zooplankton and macroinvertebrates.

From an ecological perspective, the rapid ingestion and elimination observed here indicate that rotifers may contribute to short-term redistribution of microplastics within the water column rather than their long-term bioaccumulation. Their high filtration activity and central role in planktonic food webs position them as efficient but temporary carriers of microplastics, potentially facilitating the transfer of MPs to higher consumers under natural feeding conditions. Nevertheless, environmental variables such as temperature, biofilm formation, and chemical contaminants could alter these dynamics and merit future investigation (Horie et al., 2024).

## Conclusions

This study demonstrates that both *Brachionus caudatus* and *Asplanchna brightwellii* readily ingest (1 µm) polystyrene microspheres but eliminate them efficiently within 24–48 h. The rapid depuration observed at both trophic levels, coupled with the absence of long-term tissue retention, indicates trophic transfer without classical bioaccumulation. These findings provide evidence that, in short planktonic food chains, microplastics (MPs) behave as transient contaminants rather than persistent bioaccumulative pollutants. However, our results do not allow us to exclude the possibility that, under chronic exposure, continuous ingestion of MPs leads to a state of functional persistence within the digestive tract. This implies that top predators in micro-scale food webs are consistently exposed to higher concentrations of plastic-associated stressors, such as chemical leachates, physical abrasion, and the metabolic costs of processing non-nutritive particles than primary consumers, due to the magnification of the internal dose during predation. Consequently, rotifers, through their continuous feeding and egestion activity, contribute to the dynamic redistribution of MPs in aquatic systems. Understanding these rapid ingestion–egestion dynamics is essential for assessing ecological risks in freshwater ecosystems, where exposure is chronic. However, retention is low, limiting massive bioaccumulation while allowing for significant transient exposure to toxic plastic additives (Horie et al., 2024).

Our work, however, represents only an initial step reflecting a specific model scenario using 1 µm polystyrene spheres and short-term exposure. It is crucial to emphasize that these results pertain specifically to the studied species, *B. caudatus* and *A. brightwellii*. Given the high taxonomic and ecological diversity of rotifers, different species may exhibit distinct feeding behaviors, particle size preferences, and overall physiological sensitivities to microplastic exposure. Therefore, the extrapolation of these findings to natural systems requires caution. In the environment, microplastics vary widely in size, shape, polymer type, and surface characteristics, and natural populations are typically subjected to chronic rather than acute exposure; these variables may alter ingestion, retention, and transfer dynamics (De Sá et al., 2018; Yardy et al., 2022; Zhang et al., 2023). Furthermore, it remains unclear whether egestion dynamics differ in predator–prey dynamics when organic food is absent, as constant feeding may mechanically aid the rapid clearance of particles from the gut. Moreover, environmental variables such as temperature, pH, and the presence of co-contaminants or biofilms could modify particle behavior and biological responses. Additionally, in recent work (Zhao et al., 2024), ingestion of primary microplastics by rotifers has been observed to result in fragmentation into secondary nanoplastics, which could even contribute to the persistence of these pollutants in food webs. This mechanism should be evaluated in this type of work, especially using the *Asplanchna-Brachionus* model. Future investigations must systematically address these variables, prioritizing the evaluation of microplastic retention under varying degrees of food availability, alongside the synergistic effects of particle morphology, environmental stressors, and chemical mixtures on microplastic fate in food webs. For instance, exposure to multiple co-contaminants can exacerbate physiological stress in zooplankton even at low microplastic concentrations (Sun et al., 2025); such combined toxicity could impair prey mobility and increase their susceptibility to predation, thereby inadvertently accelerating the trophic transfer of microplastics to higher consumers. Future studies should explore how these particles might interfere with specific prey defensive responses under diverse predator–prey scenarios (Liu et al., 2022; Sarma et al., 2007).

## Supplementary Information

Below is the link to the electronic supplementary material.

Supplementary file1 (DOCX 180 kb)

## Data Availability

The original data presented in this study are available from the corresponding author upon reasonable request. The code used for the analysis is provided in the Supplementary Material.

## Data Availability

The original data presented in this study are available from the corresponding author upon reasonable request. The code used for the analysis is provided in the Supplementary Material.

## Declarations

All authors have read, understood, and have complied as applicable with the statement on Ethical responsibilities of Authors as found in the Instructions for Authors.
