# Supplementary material for: Trophic transfer and egestion dynamics of microplastics in the Brachionus-Asplanchna rotifer system
Source: Environ Monit Assess. 2026 May 22;198(6):635. doi: 10.1007/s10661-026-15485-w (PMC13197250; doi:10.1007/s10661-026-15485-w)
Supplement: Supplementary file 1 — Supplementary file1 (DOCX 180 kb) [file 10661_2026_15485_MOESM1_ESM.docx]

**Supplementary Information**

The supplementary material provides additional details supporting the statistical analyses presented in the manuscript. It includes diagnostic plots of model adequacy for the Gamma GLM (Fig. S1), the results of the non-parametric Kruskal–Wallis and Dunn’s post-hoc analyses used to complement the parametric approach (Fig. S2), and the complete Tukey post-hoc comparison results associated with the Gamma GLM (Table S1). The R script used to perform all statistical analyses is also included to ensure transparency and reproducibility.


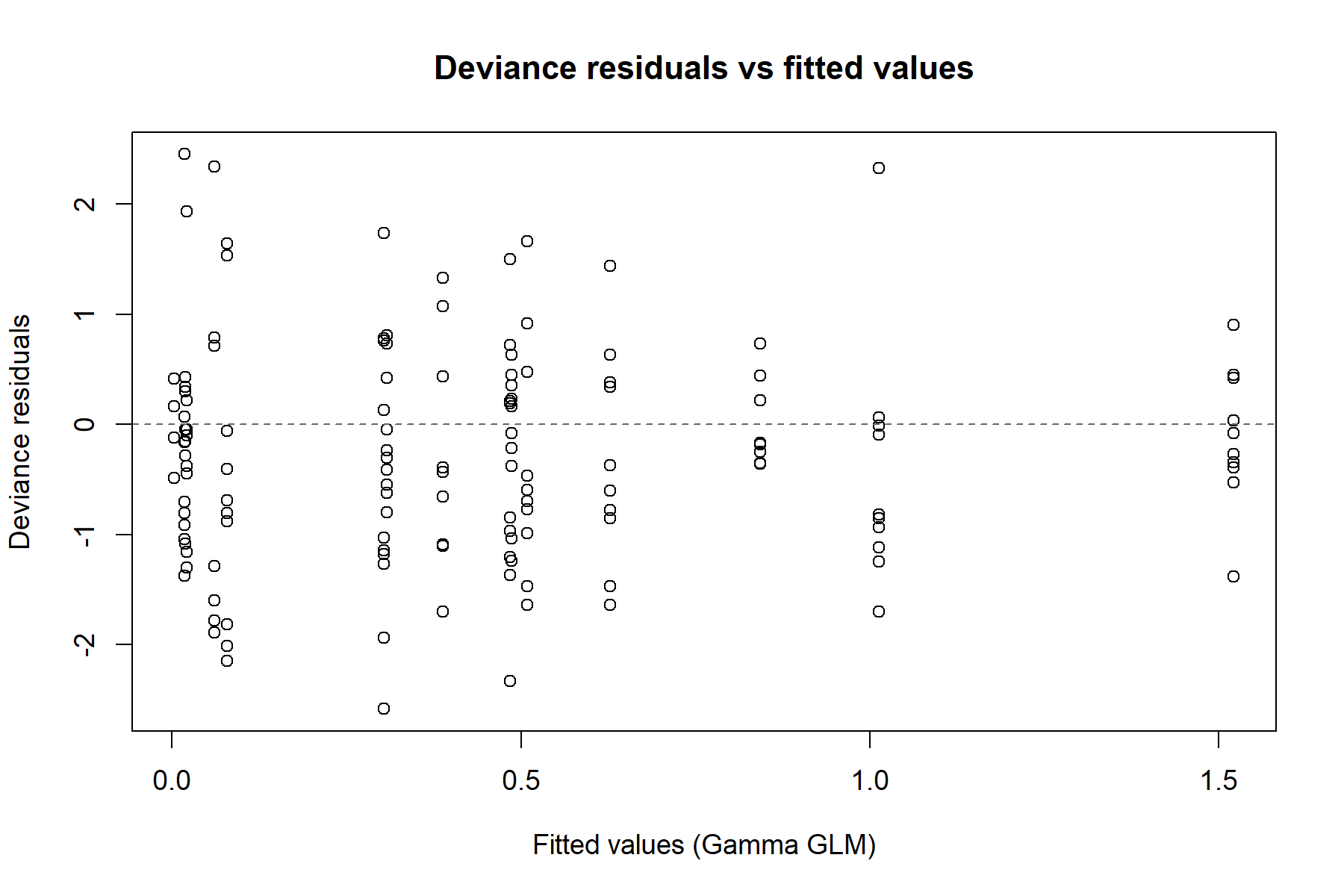


Figure S1. Plot of deviance residuals versus fitted values, confirming the absence of systematic patterns or heteroscedasticity for the GLM analysis.


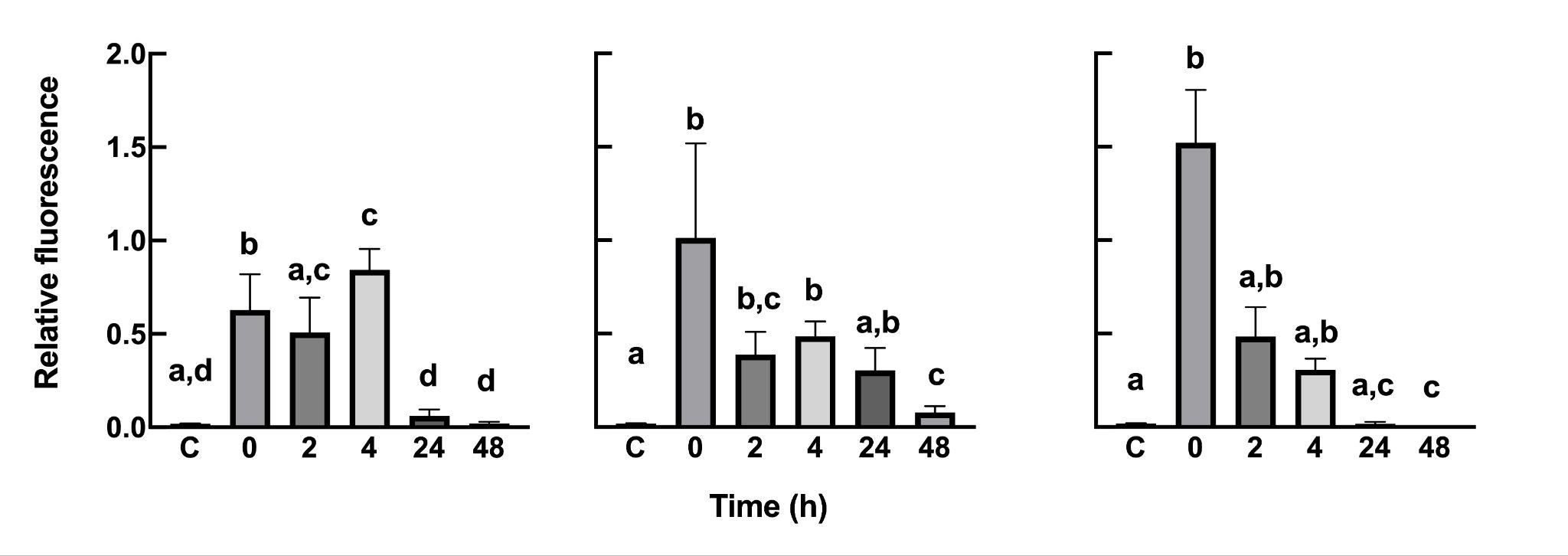


Figure S2. Kruskal–Wallis and Dunn’s post-hoc analyses of changes in relative fluorescence during the depuration period (48 h) in *B. caudatus*. a: 0.1 µg ml^-1^; b: 0.5 µg ml^-1^; c: 1.0 µg ml^-1^.

Table S1. Results of Tukey post-hoc comparisons identifying significant differences in microplastic ingestion and egestion patterns across time (hours) points and concentrations (Conc.).

| contrast-hours | Conc. | ratio | SE | df | null | t-ratio | p-value |
| --- | --- | --- | --- | --- | --- | --- | --- |
| C / 0 | 0.1 | 0.0299 | 0.0139 | 162 | 1 | -7.5630 | **4.18E-11** |
| C / 2 | 0.1 | 0.0370 | 0.0171 | 162 | 1 | -7.1088 | **5.30E-10** |
| C / 4 | 0.1 | 0.0223 | 0.0103 | 162 | 1 | -8.1971 | **1.23E-12** |
| C / 24 | 0.1 | 0.3072 | 0.1425 | 162 | 1 | -2.5441 | 1.18E-01 |
| C / 48 | 0.1 | 0.8952 | 0.4153 | 162 | 1 | -0.2385 | 1.00E+00 |
| 0 / 2 | 0.1 | 1.2345 | 0.5727 | 162 | 1 | 0.4541 | 9.98E-01 |
| 0 / 4 | 0.1 | 0.7451 | 0.3457 | 162 | 1 | -0.6341 | 9.88E-01 |
| 0 / 24 | 0.1 | 10.2614 | 4.7606 | 162 | 1 | 5.0189 | **1.99E-05** |
| 0 / 48 | 0.1 | 29.9048 | 13.8737 | 162 | 1 | 7.3244 | **1.60E-10** |
| 2 / 4 | 0.1 | 0.6036 | 0.2800 | 162 | 1 | -1.0882 | 8.85E-01 |
| 2 / 24 | 0.1 | 8.3121 | 3.8562 | 162 | 1 | 4.5647 | **1.42E-04** |
| 2 / 48 | 0.1 | 24.2238 | 11.2381 | 162 | 1 | 6.8703 | **1.96E-09** |
| 4 / 24 | 0.1 | 13.7712 | 6.3889 | 162 | 1 | 5.6530 | **1.03E-06** |
| 4 / 48 | 0.1 | 40.1333 | 18.6190 | 162 | 1 | 7.9586 | **4.45E-12** |
| 24 / 48 | 0.1 | 2.9143 | 1.3520 | 162 | 1 | 2.3056 | 1.98E-01 |
| C / 0 | 0.5 | 0.0186 | 0.0086 | 162 | 1 | -8.5927 | **2.67E-13** |
| C / 2 | 0.5 | 0.0485 | 0.0225 | 162 | 1 | -6.5239 | **1.25E-08** |
| C / 4 | 0.5 | 0.0387 | 0.0179 | 162 | 1 | -7.0113 | **9.07E-10** |
| C / 24 | 0.5 | 0.0619 | 0.0287 | 162 | 1 | -5.9963 | **1.90E-07** |
| C / 48 | 0.5 | 0.2386 | 0.1107 | 162 | 1 | -3.0890 | **2.81E-02** |
| 0 / 2 | 0.5 | 2.6111 | 1.2114 | 162 | 1 | 2.0688 | 3.09E-01 |
| 0 / 4 | 0.5 | 2.0827 | 0.9662 | 162 | 1 | 1.5814 | 6.12E-01 |
| 0 / 24 | 0.5 | 3.3353 | 1.5473 | 162 | 1 | 2.5964 | 1.04E-01 |
| 0 / 48 | 0.5 | 12.8503 | 5.9616 | 162 | 1 | 5.5038 | **2.12E-06** |
| 2 / 4 | 0.5 | 0.7976 | 0.3700 | 162 | 1 | -0.4874 | 9.97E-01 |
| 2 / 24 | 0.5 | 1.2773 | 0.5926 | 162 | 1 | 0.5276 | 9.95E-01 |
| 2 / 48 | 0.5 | 4.9213 | 2.2831 | 162 | 1 | 3.4350 | **9.64E-03** |
| 4 / 24 | 0.5 | 1.6014 | 0.7430 | 162 | 1 | 1.0150 | 9.12E-01 |
| 4 / 48 | 0.5 | 6.1701 | 2.8625 | 162 | 1 | 3.9224 | **1.77E-03** |
| 24 / 48 | 0.5 | 3.8528 | 1.7874 | 162 | 1 | 2.9073 | **4.69E-02** |
| C / 0 | 1 | 0.0124 | 0.0057 | 162 | 1 | -9.4701 | **1.65E-13** |
| C / 2 | 1 | 0.0388 | 0.0180 | 162 | 1 | -7.0020 | **9.54E-10** |
| C / 4 | 1 | 0.0611 | 0.0284 | 162 | 1 | -6.0245 | **1.65E-07** |
| C / 24 | 1 | 1.0621 | 0.4928 | 162 | 1 | 0.1300 | 1.00E+00 |
| C / 48 | 1 | 5.5294 | 2.5653 | 162 | 1 | 3.6861 | **4.14E-03** |
| 0 / 2 | 1 | 3.1425 | 1.4579 | 162 | 1 | 2.4681 | 1.40E-01 |
| 0 / 4 | 1 | 4.9457 | 2.2945 | 162 | 1 | 3.4456 | **9.31E-03** |
| 0 / 24 | 1 | 85.9492 | 39.8743 | 162 | 1 | 9.6001 | **1.65E-13** |
| 0 / 48 | 1 | 447.4412 | 207.5812 | 162 | 1 | 13.1562 | **1.08E-13** |
| 2 / 4 | 1 | 1.5738 | 0.7301 | 162 | 1 | 0.9775 | 9.24E-01 |
| 2 / 24 | 1 | 27.3503 | 12.6886 | 162 | 1 | 7.1320 | **4.67E-10** |
| 2 / 48 | 1 | 142.3824 | 66.0554 | 162 | 1 | 10.6881 | **1.52E-13** |
| 4 / 24 | 1 | 17.3785 | 8.0624 | 162 | 1 | 6.1545 | **8.51E-08** |
| 4 / 48 | 1 | 90.4706 | 41.9720 | 162 | 1 | 9.7106 | **1.63E-13** |
| 24 / 48 | 1 | 5.2059 | 2.4152 | 162 | 1 | 3.5561 | **6.46E-03** |
| C=control |  |  |  |  |  |  |  |

Script S1. R code for statistical analyses.

############################################################

# GLM (Gamma, log) for Brachionus — Control as Time level

# Excel layout: [Conc 0.1] [C_0.1] [0] [2] [4] [24] [48] | repeat for 0.5, 1.0

# Outputs: diagnostics, LRT (Type III-like), emmeans (time vs control),

# plots (raw mean±SE and GLM-fitted).

############################################################

suppressPackageStartupMessages({

library(readxl)

library(dplyr)

library(tidyr)

library(stringr)

library(ggplot2)

library(car)

library(emmeans)

library(readr)

library(forcats)

})

# ---- 1) I/O ----

infile <- "Datos_MPs_Brachionus.xlsx"

insheet <- "Hoja1"

outdir <- "outputs_glm_brachionus"

if (!dir.exists(outdir)) dir.create(outdir)

# ---- 2) Read ----

raw <- read_excel(infile, sheet = insheet)

# Optional: drop any header rows that contain "Conc" in the first column

first_col <- names(raw)[1]

raw <- raw %>% filter(!grepl("^\\s*Conc", .data[[first_col]], ignore.case = TRUE))

# ---- 3) Helpers to match your exact layout ----

# Controls look like: C_0.1, C_0.5, C_1.0

control_cols <- grep("^C(_|\\.)\\d+\\.?\\d*$", names(raw), value = TRUE)

if (length(control_cols) == 0) stop("No control columns (C_*) were found. Check headers.")

# Build a long block from each control + the next 5 time columns

# The 5 time columns immediately FOLLOW the C_* column in your file

TIME_LEVELS <- c("0","2","4","24","48")

build_block <- function(df, ctrl_name, n_times = 5) {

cnames <- names(df)

idx <- which(cnames == ctrl_name)

if (length(idx) != 1) stop("Control column not found uniquely: ", ctrl_name)

t_idx <- seq.int(idx + 1, idx + n_times)

if (max(t_idx) > length(cnames))

stop("Expected five time columns after ", ctrl_name, " but file ended.")

time_cols <- cnames[t_idx]

# For printing/debug

message("Block for ", ctrl_name, " uses time columns: ",

paste(time_cols, collapse = ", "))

# Pivot: control + its 5 time columns

df %>%

select(all_of(c(ctrl_name, time_cols))) %>%

pivot_longer(everything(), names_to = "Name", values_to = "Fluor") %>%

mutate(

Fluor = suppressWarnings(as.numeric(Fluor)),

# Strip any ".1", ".2" suffix to reveal the base header (0,2,4,24,48)

base_name = sub("\\..*$", "", Name),

Time_label = if_else(Name == ctrl_name, "C", base_name),

Conc = suppressWarnings(as.numeric(sub("^C[_\\.]", "", ctrl_name)))

) %>%

select(Conc, Time_label, Fluor)

}

# Bind all three blocks

long <- bind_rows(lapply(control_cols, \(c) build_block(raw, c))) %>%

filter(!is.na(Fluor), !is.na(Conc), Time_label %in% c("C", TIME_LEVELS)) %>%

mutate(

Time_f = factor(Time_label, levels = c("C", TIME_LEVELS), ordered = TRUE),

Conc_f = factor(Conc, levels = c(0.1, 0.5, 1.0), ordered = TRUE)

)

# Design check

design_tab <- as.data.frame(with(long, table(Time_f, Conc_f)))

write_csv(design_tab, file.path(outdir, "design_table_Time_by_Conc.csv"))

print(with(long, table(Time_f, Conc_f)))

# ---- 4) GLM (Gamma, log) ----

glm_fit <- glm(Fluor ~ Time_f * Conc_f, data = long, family = Gamma(link = "log"))

# ---- 5) Diagnostics ----

png(file.path(outdir, "diagnostic_residuals_vs_fitted.png"),

width = 1800, height = 1200, res = 220)

plot(fitted(glm_fit), residuals(glm_fit, type = "deviance"),

xlab = "Fitted values (Gamma GLM)",

ylab = "Deviance residuals",

main = "Deviance residuals vs fitted values")

abline(h = 0, lty = 2, col = "gray40")

dev.off()

# ---- 6) Type III-like LRT via nested models ----

m_full <- glm_fit

m_no_int <- update(m_full, . ~ . - Time_f:Conc_f)

m_no_time <- glm(Fluor ~ Conc_f, data = long, family = Gamma(link="log"))

m_no_conc <- glm(Fluor ~ Time_f, data = long, family = Gamma(link="log"))

lrt_int <- anova(m_no_int, m_full, test = "LRT")

lrt_time <- anova(m_no_time, m_no_int, test = "LRT")

lrt_conc <- anova(m_no_conc, m_no_int, test = "LRT")

collect_lrt <- function(lrt_obj, effect) {

data.frame(

Effect = effect,

ChiSq = as.numeric(lrt_obj$Deviance[2]),

df = as.numeric(lrt_obj$Df[2]),

p_value = as.numeric(lrt_obj$`Pr(>Chi)`[2])

)

}

anova_tidy <- bind_rows(

collect_lrt(lrt_int, "Time_f:Conc_f"),

collect_lrt(lrt_time, "Time_f"),

collect_lrt(lrt_conc, "Conc_f")

)

write_csv(anova_tidy, file.path(outdir, "glm_typeIII_LR_nested.csv"))

print(anova_tidy)

# ---- 7) emmeans: Time vs Control within each Concentration ----

emm_time_by_conc <- emmeans(glm_fit, ~ Time_f | Conc_f, type = "response")

# Compare each time (0,2,4,24,48) vs 'C' in the SAME concentration

vs_control <- contrast(emm_time_by_conc, method = "trt.vs.ctrl", ref = "C")

write.csv(as.data.frame(emm_time_by_conc),

file.path(outdir, "emmeans_Time_by_Conc_response.csv"), row.names = FALSE)

write.csv(as.data.frame(vs_control),

file.path(outdir, "posthoc_Time_vs_Control_within_Conc.csv"), row.names = FALSE)

# Optional: Tukey among times within each concentration

pairs_time <- contrast(emm_time_by_conc, method = "tukey")

write.csv(as.data.frame(pairs_time),

file.path(outdir, "posthoc_Tukey_Time_within_Conc.csv"), row.names = FALSE)

# ---- 8) Descriptive plot (mean ± SE) including 'C' ----

summary_se <- long %>%

mutate(Time_num = as.numeric(fct_relevel(Time_f, "C","0","2","4","24","48"))) %>%

group_by(Time_f, Time_num, Conc_f) %>%

summarise(n = n(),

mean = mean(Fluor, na.rm = TRUE),

sd = sd(Fluor, na.rm = TRUE),

se = sd / sqrt(n),

.groups = "drop")

p <- ggplot(summary_se, aes(x = Time_num, y = mean, group = Conc_f)) +

geom_ribbon(aes(ymin = mean - se, ymax = mean + se, fill = Conc_f), alpha = 0.25) +

geom_line(aes(color = Conc_f), linewidth = 1) +

geom_point(aes(color = Conc_f), size = 2) +

scale_x_continuous(breaks = 1:6, labels = c("C","0","2","4","24","48")) +

labs(x = "Time ('C' = Control)",

y = "Fluorescence (mean ± SE)",

color = "Concentration", fill = "Concentration",

title = "Fluorescence by time within concentration (Control included)") +

theme_minimal(base_size = 12) +

theme(legend.position = "right", panel.grid.minor = element_blank())

ggsave(file.path(outdir, "line_SE_ribbons_including_ControlAsTime.png"),

plot = p, width = 8, height = 5, dpi = 300)

# ---- 9) Model-based fitted means (±95% CI) ----

emm_df <- as.data.frame(emm_time_by_conc) %>%

mutate(Time_num = as.numeric(fct_relevel(Time_f, "C","0","2","4","24","48")))

p_fit <- ggplot(emm_df, aes(x = Time_num, y = response, group = Conc_f)) +

geom_ribbon(aes(ymin = lower.CL, ymax = upper.CL, fill = Conc_f), alpha = 0.25) +

geom_line(aes(color = Conc_f), linewidth = 1) +

geom_point(aes(color = Conc_f), size = 2) +

scale_x_continuous(breaks = 1:6, labels = c("C","0","2","4","24","48")) +

labs(x = "Time ('C' = Control)",

y = "Fitted mean (±95% CI)",

color = "Concentration", fill = "Concentration",

title = "GLM-fitted means by time within concentration") +

theme_minimal(base_size = 12) +

theme(legend.position = "right", panel.grid.minor = element_blank())

ggsave(file.path(outdir, "line_GLMFitted_95CI_including_ControlAsTime.png"),

plot = p_fit, width = 8, height = 5, dpi = 300)

# ---- 10) Session info ----

sink(file.path(outdir, "sessionInfo.txt")); print(sessionInfo()); sink()

message("Done. Outputs written to: ", normalizePath(outdir))
